# Longevity of antibody responses is associated with distinct antigen-specific B cell subsets early after infection

**DOI:** 10.3389/fimmu.2024.1505719

**Published:** 2024-12-17

**Authors:** Lisan H. Kuijper, Christine Kreher, George Elias, Mathieu Claireaux, Gius Kerster, Amélie V. Bos, Mariël C. Duurland, Veronique A. L. Konijn, Alberta G. A. Paul, Nina de Jong, Rivka de Jongh, Maurice Steenhuis, Juan J. Garcia-Vallejo, Marit J. van Gils, Taco W. Kuijpers, Filip Eftimov, Theo Rispens, C. Ellen van der Schoot, S. Marieke van Ham, Anja ten Brinke

**Affiliations:** ^1^ Sanquin Research and Landsteiner Laboratory, Amsterdam UMC, University of Amsterdam, Amsterdam, Netherlands; ^2^ Amsterdam Institute for Immunology and Infectious Diseases, Amsterdam, Netherlands; ^3^ Department of Medical Microbiology and Infection Prevention, Laboratory of Experimental Virology, Amsterdam UMC Location University of Amsterdam, Amsterdam, Netherlands; ^4^ Cytek Biosciences, Inc., Fremont, CA, United States; ^5^ Department of Molecular Cell Biology and Immunology, Amsterdam Infection & Immunity and Cancer Center Amsterdam, Amsterdam University Medical Centers, Free University of Amsterdam, Amsterdam, Netherlands; ^6^ Department of Pediatric Immunology, Rheumatology and Infectious Diseases, Emma Children’s Hospital, Amsterdam University Medical Centers, University of Amsterdam, Amsterdam, Netherlands; ^7^ Department of Neurology, Amsterdam University Medical Center, University of Amsterdam, Amsterdam, Netherlands; ^8^ Swammerdam Institute for Life Sciences, University of Amsterdam, Amsterdam, Netherlands

**Keywords:** declining/sustained antibody titers, deep-phenotyping, SARS-CoV-2, CD4+ T cells, neutralization

## Abstract

**Introduction:**

Upon infection, T cell-driven B cell responses in GC reactions induce memory B cells and antibody-secreting cells that secrete protective antibodies. How formation of specifically long-lived plasma cells is regulated via the interplay between specific B and CD4+ T cells is not well understood. Generally, antibody levels decline over time after clearance of the primary infection.

**Method:**

In this study, convalescent individuals with stable RBD antibody levels (n=14, “sustainers”) were compared with donors (n=13) with the greatest antibody decline from a cohort of 132. To investigate the role of the cellular immune compartment in the maintenance of antibody levels, SARS-CoV-2-specific responses at 4 to 6 weeks post-mild COVID-19 infection were characterized using deep immune profiling.

**Results:**

Both groups had similar frequencies of total SARS-CoV-2-specific B and CD4+ T cells. Sustainers had fewer Spike-specific IgG+ memory B cells early after infection and increased neutralizing capacity of RBD antibodies over time, unlike the declining group. However, declining IgG titers correlated with lower frequency of Spike-specific CD4+ T cells.

**Conclusion:**

These data suggest that “sustainers” have unique dynamics of GC reactions, yield different outputs of terminally differentiating cells, and improve the quality of protective antibodies over time. This study helps identify factors controlling formation of long-lived PC and sustained antibody responses.

## Introduction

Establishment of sustained protective antibody levels is important for clearance of pathogens and protection against reinfection. Activated B cells differentiate via extrafollicular and germinal center (GC) pathways into antigen-specific plasmablasts (PB) and plasma cells (PC) that secrete protective antibodies. Whereas PB are relatively short-lived, PC that home to the bone marrow often survive for decades while continuously secreting antibodies, thereby establishing long-term humoral immune protection against subsequent infections (1). It remains highly relevant to understand how formation of long-term humoral immune protection is regulated. Our group and others have described that, upon severe acute respiratory syndrome coronavirus 2 (SARS-CoV-2) infection, neutralizing antibodies produced against Spike or its receptor binding domain are needed to confer immune protection from symptomatic SARS-CoV-2 infection (2–6). These studies also showed that receptor-binding domain (RBD)-IgG levels exhibit a two-phase decay profile with a rapid decline over the first 4 months and a more gradual decline after that (7–9). Presumably, the rapid decline in antibody response in the first 6 months after initial exposure to the virus is related to loss of short-lived PBs, whereas long-lived PC are responsible for maintaining a basal level of antibodies that persist at later time points (10). Indeed, SARS-CoV-2-specific PC were still found 7 months after infection in the bone marrow and detected until at least 11 months in some individuals, suggesting a stable maintenance of antibody levels in late convalescence through these long-lived PC (11). However, other studies have suggested a possible lack of Spike-specific long-lived PC generation after infection (12) and vaccination (13, 14) in the bone marrow.

Long-term humoral immunity relies on formation of GCs in lymph nodes. In these GC reactions, memory B cells (MBCs) and long-lived PC develop, producing a high-affinity antibody repertoire through somatic hypermutation of the immunoglobulin-variable region. SARS-CoV-2-specific MBC frequencies increase in the first 3 to 4 months following infection. MBCs can differentiate upon antigenic rechallenge into antibody-secreting cells that are mostly of the short-lived PB phenotype (15–17). Longitudinal analysis of SARS-CoV-2-specific MBC clones showed progressive acquisition of mutations over 6 months, pointing to an ongoing GC response (18). In addition, SARS-CoV-2-specific MBCs evolving during late convalescence expressed antibodies with increased neutralizing capacity and breadth compared with earlier time points supporting long-term persistence of GCs (16, 19). Ongoing GC reactions and selection of B cell receptor (BCR) variants with the highest affinity out of the large pool of antigen-specific GC B cells requires help from T follicular helper cells (Tfh) to allow reentry of the differentiating B cells into the GC dark zone for further proliferation. During proliferation, mutations are continuously introduced enhancing the probability of acquiring a higher affinity BCR, which will result in a greater share of help from Tfh cells and thus driving positive selection. SARS-CoV-2-specific Tfh cells were found in lymphoid tissues and sites of infection in convalescent individuals for months after infection pointing to robust GC responses (20). In support, the frequency of SARS-CoV-2-specific Tfh memory cells in circulation appeared to be stable up to 6 months post-infection (15, 17, 21).

SARS-CoV-2 infection in healthy naïve individuals generated the opportunity to study the memory formation of immune responses and identify correlates of durable humoral immunity. In our group, immune responses against SARS-CoV-2 have been studied in convalescent and vaccinated cohorts (22–28). Notably, it was observed by us and others that antibody kinetics varied among convalescent donors, with some donors having a very quick decay of RBD antibodies, whereas some had more stable antibody levels in the same time period (7, 29, 30). Individuals with detectable antibody levels over time are protected against reinfection (31–33). Still, the mechanism that controls formation of long-lived PC maintaining high antibody levels over time is poorly understood. Recently, we identified specific B cells at different (precursor) stages of B cell differentiation into MBCs and antibody-secreting cells by deep profiling of SARS-CoV-2-specific B cell responses in different disease severities of COVID-19 (28).

To gain insight in which immune cell populations might be involved in sustained and/or declining antibody responses, we performed deep profiling of SARS-CoV-2-specific B- and T cell populations in blood-derived PBMCs of individuals with either rapidly declining (DAb) or sustained (SAb) anti-RBD IgG titers after infection. Interestingly, early after infection, the output of IgG+ MBC in the blood was less in the SAb group, whereas only in this group did the neutralization potency of anti-RBD IgG antibodies increase significantly over 5 months. These findings indicate that donors with sustained antibody responses are more prone to ongoing GC responses whereas donors with declining antibodies seem to be more constrained toward memory B cell output early after infection.

## Methods

### Study participants and design

The study is in accordance with the declaration of Helsinki and according to Dutch regulations. Data and samples were collected only from voluntary, non-remunerated, adult donors who provided written informed consent as part of routine blood collection procedures of the Sanquin Blood Supply Foundation (Blood Bank). The study was approved by the Ethics Advisory Council of Sanquin Blood Supply Foundation. Donors having recovered from mild convalescent COVID-19 disease were identified at Sanquin Blood Bank, Amsterdam, the Netherlands (410 donors). The participants were further selected on availability of more than two frozen PBMC vials at the early time point, two or more serological measurements, and more than 49 days between the first and last time points of serology (132 donors). For these selected donors, all available plasma samples were measured at least twice to determine RBD-IgG titers as described below and donors with signs of reinfection were excluded. Antibody levels show exponential decay over time; therefore, we assumed that the decay in log-transformed antibody levels was linear to estimate the half-life. For each donor, a linear model was fitted on the log-transformed antibody levels, with time expressed as days post symptom onset (PSO) as described before (34). The estimated slope of the model was used to calculate the donor’s antibodies’ half-life in days: $t_{1/2}=\log\left(\frac{1}{2}\right)/slope$ . The top 10% with shortest half-life (DAb, n=13) and top 10% with longest half-life, including increasing titers (SAb, n=14), were selected for this study.

### Isotype-specific antibody ELISA

IgM, IgG, and IgA to RBD and nucleocapsid (NC) were measured as described previously (7). RBD and NC proteins were produced as described before (7). Pooled convalescent plasma or serum was included on each plate as a calibrator (set to a value of 100 AU/mL) to quantify the signals. Results were expressed as arbitrary units (AU) per mL (AU/mL) and represent a semiquantitative measure of the titer of IgG, IgA, and IgM antibodies to RBD and NC.

### Neutralization tested by competitive ELISA

Serum samples from the time point of PBMC sampling (T1) and the last time point of sampling (Tfinal) were tested for the neutralizing capacity of their SARS‐CoV‐2 antibodies using a competitive assay as described previously (7). The IC50 value was calculated by using a non-linear fitting model with a restrained slope of −1 and weighted by 1/Y^2^ on the output of dilution factor versus the percentage of non-inhibition. By multiplying the calculated IC50 value with the RBD titer of the same serum sample, we excluded the influence of possible variations in the RBD titer between individuals.

### Peripheral blood mononuclear cells

Peripheral blood mononuclear cells (PBMC) were isolated by Ficoll Paque Plus gradient separation (GE Healthcare, Chicago, IL, USA) and stored in 10% dimethyl sulfoxide in fetal bovine serum (Thermo Fisher Scientific, Waltham, MA, USA) for future use. All procedures were in accordance with guidelines established by the Sanquin Medical Ethical Committee and in line with the Declaration of Helsinki.

### Protein design and preparation for detection of antigen-specific B cells

SARS-CoV-2 S-2P, RBD, influenza A hemagglutinin (H1N1pdm2009, A/Netherlands/602/2009, GenBank: CY039527.2), RSV prefusion stabilized F (DS-Cav1), and constructs with avi-tag and/or hexahistidine (his)-tag and/or strep-tag were expressed and purified as previously described (2, 28). Avi-tagged proteins were biotinylated with a BirA500 biotin-ligase reaction kit according to the manufacturer’s instruction (Avidity). Tetanus toxoid was purchased from Creative Biolabs (Vcar-Lsx003). Nucleocapsid and tetanus toxoid were aspecifically biotinylated using EZ-Link Sulfo-NHS-LC-Biotinylation Kit (Thermo Fisher) according to the manufacturer’s instructions. Biotinylated protein antigens were labeled individually with fluorescently labeled streptavidin and multimerized (BB515, BD Biosciences; BUV615, BD Biosciences; AF647, BioLegend; BV421, BioLegend), as described previously (28). Biotinylated proteins and fluorescently labeled streptavidin were mixed at a 2:1 protein to fluorochrome molar ratio and incubated at 4°C for 1 h. Unbound streptavidin conjugates were blocked with 10 µM biotin (GeneCopoiea) for at least 10 min. A combinatorial probe staining strategy was used for simultaneous identification of multiple B cell specificities in a single sample. This strategy uses multiple combinations of two fluorophores to increase the number of specificities that can be detected and decrease aspecific binding. In our study, we were able to detect six different antigen-specificities using five distinct fluorophores. Probes were labeled in the following manner: SARS-CoV-2 S (AF647, BV421), H1N1 HA (BUV615, BV421), RSV F (AF647, BUV615), NC (AF647, BB515), tetanus toxoid (BB515, BV421), and RBD (PE-Cy7). Individually labeled proteins were then equimolarly mixed and kept at 4°C before usage. A final concentration of 45.5 nM of each probe is used to label B cells.

### *Ex vivo* B cell phenotyping of antigen-specific B cells

PBMCs were thawed in Iscove’s Modified Dulbecco’s Medium (Lonza, Verviers, Belgium) with 10% fetal calf serum (Bodinco, Alkmaar, The Netherlands), 10 million PBMCs were depleted of CD3+ cells using EasySep™ Human CD3 Positive Selection Kit II and EasyPlate™ EasySep™ Magnet (STEMCELL Technologies, Vancouver, Canada), and between 0.8 and 3.5 million B cells were obtained for staining. A 29-color spectral flow cytometry panel was designed to enable comprehensive and simultaneous immunophenotyping of total B cells and B cells with antigen specificity to six proteins (**Supplementary Tables S1**, **S2**). A total of 23 colors were assigned for deep characterization of the B cell compartment, 5 colors were assigned to capture antigen specificity using double discrimination, and 2 colors were assigned to exclude dead cells and for a dump channel to remove cells expressing CD3, CD4, CD16, or CD56. LIVE/DEAD™ Fixable Blue Dead Cell Stain Kit from Invitrogen™ (Thermo Fisher Scientific, Waltham, MA, USA) was used to exclude non-viable cells. BD Horizon™ Brilliant Stain Buffer Plus (BD Biosciences, Franklin Lakes, NJ, USA) was supplemented to the staining mixture according to the manufacturer’s instructions. Cells were stained at 4°C for 30 min with the mix of multimerized proteins and the mix of fluorochrome-conjugated antibodies simultaneously. Following staining, cells were washed twice with a washing buffer containing 1% bovine serum albumin (Sigma-Aldrich, Saint Louis, USA) and 1 mM ethylenediaminetetraacetic acid (EDTA) in phosphate-buffered saline (Fresenius Kabi, ‘s-Hertogenbosch, The Netherlands), and then fixed with 1% cold paraformaldehyde for 10 min at room temperature on a shaker and then washed twice with washing buffer. Data were acquired on Cytek Aurora 5L using SpectroFlo^®^ software (Cytek Biosciences, Fremont, California, United States).

### Spectral flow cytometry data preprocessing

PBMCs were depleted of CD3+ cells and labeled with a panel of fluorochrome-conjugated antibodies to surface proteins and fluorochrome-labeled probes and acquired using spectral flow cytometry for a deep profiling of antigen-specific B cells in convalescent COVID-19 patients and healthy controls in three batches. All FCS files were gated to remove debris, doubles, and dead cells, and cells positive for CD3-, CD4-, CD16-, or CD56-positive events were excluded in a dump channel. Subsequently CD19 was used to identify B cells (gating strategy is shown in **Supplementary Figure S2A**) and a range from 101,469 to 708,000 CD19+ B (median: 239,799) cells were measured per donor. B cells specific for each of the six proteins were gated based on the combination of fluorochrome-conjugated streptavidin. RBD-specific cells were gated from the Spike-specific gate. Gated data were further processed with the R programming language (http://www.r-project.org) and Bioconductor (http://www.bioconductor.org) software. First, antigen specificity was integrated as a logical variable in the data. Anomalies in the data were removed using the flow_auto_qc function from the FlowAI package in R (35). Data were transformed with an inverse hyperbolic sine (arcsinh) transformation. Batch effects were modeled using reference samples stained and acquired with each batch to control for signal fluctuation that might occur over time due to changes in instrument performance. The model was then used to remove batch effects from the data using a normalization algorithm. Modeling of batch effect and data normalization was done using the CytoNorm package in R (36).

For initial clustering, FlowSOM was used, which is based on self‐organizing maps and hierarchical consensus meta-clustering (36). Total B cells and antigen-specific B cells were clustered into 400 nodes, using a 20 × 20 grid with the scaled expression of 23 cell surface proteins used as input. Nodes were then meta-clustered using the ConsensusClusterPlus function with k = 40 for hierarchical consensus clustering as implemented in the ConsensusClusterPlus package in R (37). A random subset of the cells in the data were then aggregated per meta-cluster and median of the unscaled expression of each cell surface protein was calculated to generate colored heatmap for meta-clusters versus cell surface proteins using the make.pheatmap function from Spectre package in R (38). Meta-clusters were then annotated, and some of the meta-clusters were merged based on visual inspection of heatmaps (where the differential expression of cell surface proteins was not biologically meaningful for some neighboring meta-clusters).

### Activation-induced marker assay to characterize SARS-CoV-2 specific T cell responses

Frozen PBMCs with median 18.6 million cells per vial (min: 8.4 million, max: 30.2 million) were thawed. Two million PBMCs were stimulated overnight at 37°C with or without SARS-CoV-2 peptide pools (PepMix™, JPT, 1 μg/ml in DMSO) in the presence of 3 μg/ml brefeldin A (eBioscience™, Thermo Fisher). Peptide pools consisted of 15-mers with an 11-amino acid overlap spanning the entire protein sequence of Spike and nucleocapsid. Spike peptides were divided into two pools (S1 and S2) and separately used for PBMC stimulation. Cells were stained with FSV575V viability dye and the chemokine markers anti-CCR6, anti-CCR4, anti-CXCR5, and anti-CXCR3; the activation markers anti-PD1, anti-HLADR, anti-CD38, and anti-ICOS; and the differentiation markers anti-CD45RA, anti-CD27, and anti-CD14 antibodies for 30 min at room temperature. Cells were subsequently fixed and permeabilized using the Foxp3/Transcription Factor Staining Buffer Set (eBioscience™, Thermo Fisher) and stained for the cytokines IL21, IL17A, IL4, IFNg, and TNF; the lineage markers CD4, CD8, and CD3; and the activation markers CD40L (BioLegend), CD137, and CD69 for 30 min at 4°C and analyzed on a BD Symphony flow cytometer. Data were analyzed by FlowJo (Tree Star). Median 287,340 CD4+ T cells (min: 188,305, max: 701,800) were measured per donor. Antibodies were purchased from BD Biosciences-Pharmingen unless otherwise stated (**Supplementary Table S3**). Unfortunately, the surface expression of the chemokine CXCR5 on activated cells was highly affected by the assay setup, and thus antigen-specific T follicular helper cells (Tfh, CXCR5 positive) could not be identified and are likely mixed in the other T helper populations. The gating strategy is shown in **Supplementary Figure S4A**.

### Statistics and data visualization

Statistics and data visualization was performed using GraphPad Prism version 9.1.1 and the programming language R, using RStudio. For visualization of marker expression, cell frequencies between groups, ggplot2 (V3.3.2), ggpubr (V0.2.5), rstatix (V0.7.0), and ggridges (V0.5.3) packages in R were used. As no normal distribution was assumed, the Wilcoxon signed-rank test was used to compare two or more groups, with unpaired and paired analysis as necessary. The results were adjusted for multiple comparisons using the Bonferroni correction as implemented in the rstatix package. The non-parametric Spearman’s rank-order correlation was used to test for correlation. We used the following convention for symbols indicating statistical significance; ns P > 0.05, *P ≤ 0.05, **P ≤ 0.01, ***P ≤ 0.001, ****P ≤ 0.0001. As there were certain B cell populations identified in the FlowSom clustering that were not present in all 21 donors measured for B cell analysis, we implemented a cutoff of minimal events to be analyzed per population of 1% of the total antigen-specific number for Spike, RBD, and NC. The distribution of antigen-specific cells per cluster was also checked between all donors to make sure all contributed to each cluster. Antigen-specific populations that fell below the cutoff were not taken along in further statistical analysis.

## Results

### Characteristics of the declining and sustained RBD-IgG antibody groups

**Figure 1:**
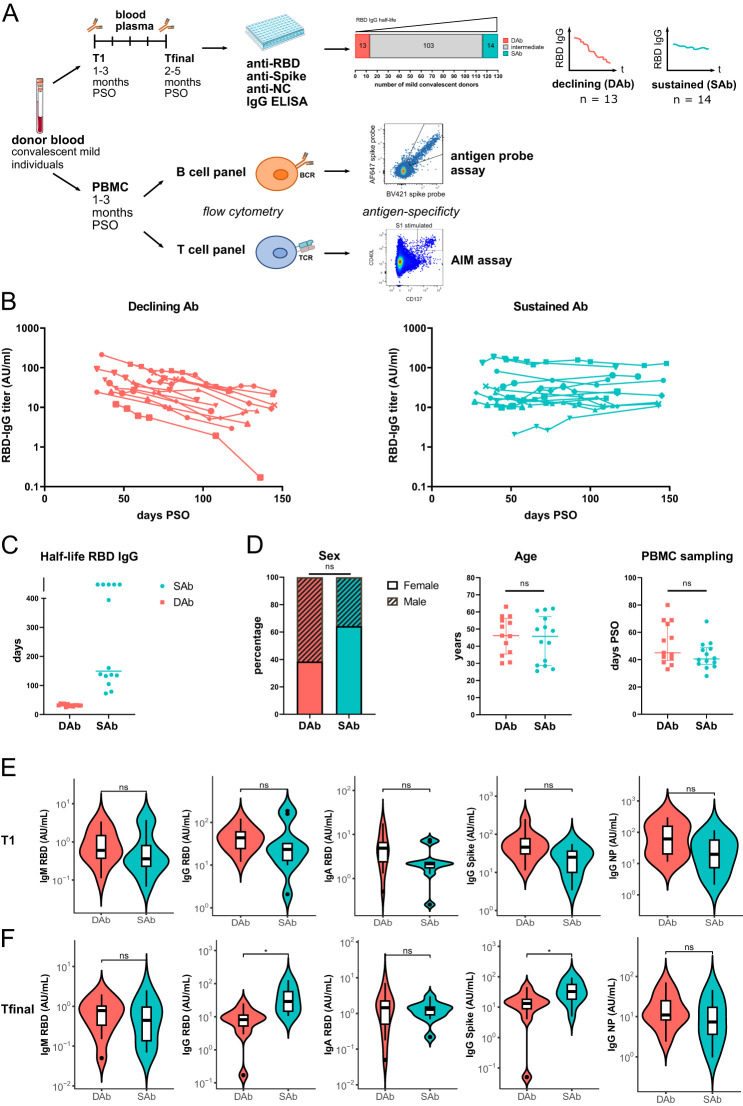
Cohort of convalescent individuals with declining or sustained RBD-IgG antibody titers. **(A)** Blood plasma from 132 mild convalescent individuals were sampled, and the anti-RBD IgG concentration was measured longitudinally up to 157 days after symptom onset (PSO) by ELISA. Groups of individuals with the shortest (DAb, n=13) or longest (SAb, n=14) anti-RBD IgG half-lives were selected for further immunophenotyping of their antigen-specific B and T cells. PBMCs from these individuals were either overnight stimulated with Spike and NC peptide pools to detect and phenotype Spike- and NC-specific T cells in an AIM-assay or CD3 depleted and stained with fluorescently labeled antigens to detect SARS-CoV-2-specific B cells. **(B)** Anti-RBD IgG titers measured up to 157 days PSO for the DAb and SAb groups. **(C)** RBD-IgG half-life of DAb and SAb donors, as estimated using linear mixed-effects modeling integrating all measuring points. In the case of rising antibody levels, half-life in days was arbitrarily assigned a value of 500. **(D)** Distribution of age, sex, and days PSO of PBMC sampling in the DAb and SAb convalescent donor groups. Individual values and median with interquartile range are shown. Mann–Whitney test was performed. **(E, F)** SARS-CoV-2-specific antibody titers of the DAb and SAb convalescent donor groups at the time point of the PBMC sampling, T1 **(E)**, and at the final time point of measurements, Tfinal **(F)**. Violin plots are shown with median and extend from the 25th to 75th percentiles. Mann–Whitney test was performed. ns, non-significant. *P < 0.05.

In this study, 410 SARS-CoV-2 experienced individuals >18 years of age were sampled for peripheral blood at the beginning of the pandemic, between 08 April 2020 and 15 September 2020, as part of a larger convalescent blood donor observational study (34). There were 132 individuals who had experienced mild infection that were selected for evaluation of RBD-IgG antibody kinetics based on presence of >2 sample points for serology up to 150 days post-symptom onset (PSO), of which first and last samples were at least 50 days apart and availability of >1 vial of PBMCs (**Figure 1A**, **Supplementary Figure S1A**). Linear mixed-effects modeling integrating all measuring points of RBD-IgG was used to calculate the half-life of RBD-IgG (34). Based on the estimated half-life of RBD-IgG, 13 individuals were selected who showed the fastest declining antibody titers (Declining Antibody group) as well as 14 individuals who showed the most stable antibody titers over time (Sustaining Antibody group) (**Figures 1A, B**). The estimated half-life for RBD-IgG was clearly different between the two groups (**Figure 1C**). The same was observed for estimated half-life for Spike-IgG and to lesser extent for NC-IgG (**Supplementary Figure S1**). The median age and sex of donors were comparable between both groups, as were the number of days PSO for the PBMC samples analyzed (**Figure 1D**). Evaluation of RBD-IgG, RBD-IgA, RBD-IgM, Spike-IgG, and NC-IgG levels at time point of PBMC sampling (T1, median: 44d [27-80]) showed no significant differences between the SAb and DAb groups (**Figure 1E**). In line with selection criteria for both groups, at the final time point, around 128 days PSO (Tfinal, median: 128d [57-148]), RBD-IgG and Spike-IgG titers were significantly higher in the SAb group, whereas RBD-IgA, RBD-IgM, and NC-IgG levels were similar between the SAb and DAb groups (**Figure 1F**).

### Lower frequency of IgG+ memory B cells in the Spike-specific B cell pool in group with sustained antibody responses

**Figure 2:**
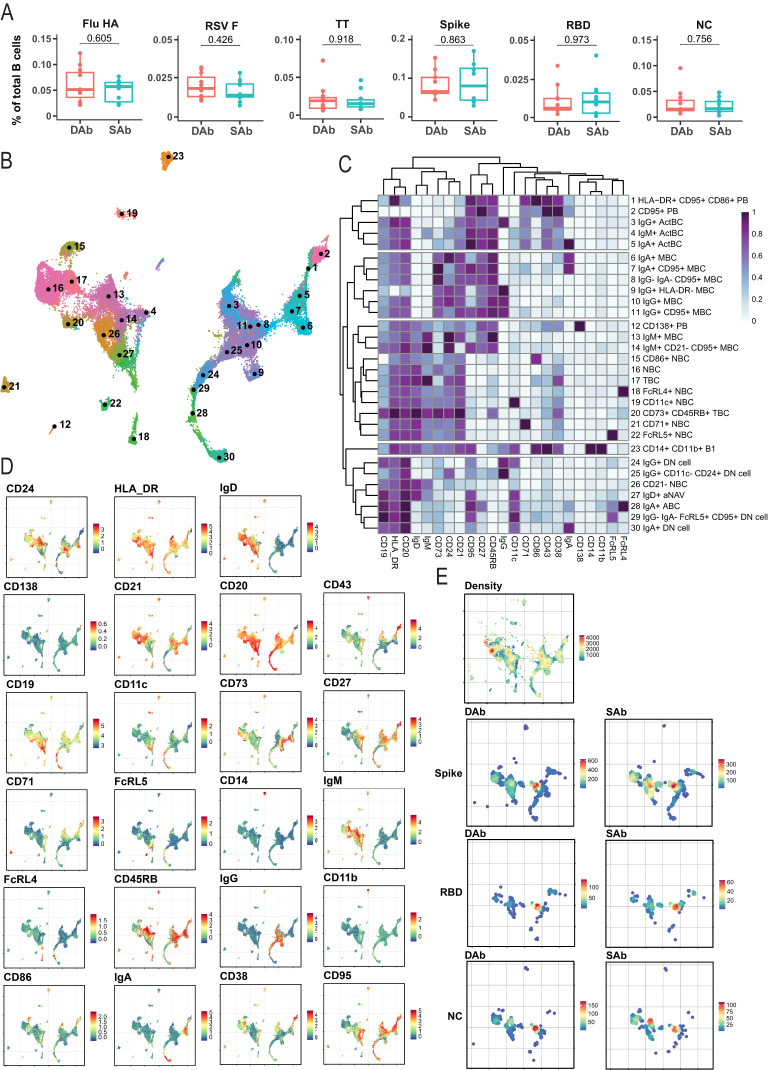
Immune phenotyping of SARS-CoV-2-specific B cells. B cells were analyzed by spectral flow cytometry for DAb (n=11) cohorts and SAb (n=10). **(A)** Frequency of antigen-specific B cells out of total CD19+ B cells for DAb and SAb groups. Mann–Whitney test was performed. **(B)** UMAP overview of numbered clusters is shown. **(C)** Heatmap with the relative protein marker expression for each annotated population with corresponding number on the UMAP. **(D)** Protein marker expression of all measured markers on B cells plotted as UMAP distribution. **(E)** UMAP distribution of all included cells (Density) and equal number of antigen-specific B cells in the DAb and SAb cohorts for Spike, RBD, and NC SARS-CoV-2 antigens.

Next, to investigate if differences in antibody RBD-IgG decay over time are associated with formation of specific SARS-CoV-2 B cell subsets, deep profiling of the SARS-CoV-2-specific B cell compartment in blood was performed relatively early after infection, around 45 days PSO (T1). In addition to the three SARS-CoV-2 antigen specificities (Spike, RBD, and NC), B cells reactive to hemagglutinin (HA) from H1N1/pdm2009 influenza virus, fusion glycoprotein (F) from respiratory syncytial virus (RSV), or tetanus toxoid vaccine (TT) were evaluated to compare the composition of the specific B cell compartments early after infection (SARS-CoV-2) to those specific B cell compartments (Flu/RSV/TT) that were established longer ago. Of note, during the time period of analysis, no influenza or RSV infections occurred and due to travel restrictions, almost no adult received a TT vaccination. No differences were observed between the SAb and DAb groups in frequency of Flu-HA, RSV-F, and TT specific total B cells (**Figure 2A**). In addition, the frequency of SARS-CoV-2-specific B cells (Spike, RBD, and NC) was similar between the DAb and SAb groups (**Figure 2A**). With respect to RBD, these results indicate that the size of the circulating RBD-specific B cell compartment at 45 days PSO is not indicative for the measured differences in anti-RBD titer decay over time.

In order to analyze the different differentiation and activation stadia of the antigen-specific B cell compartments, a total of 25 unique flow cytometry markers (**Supplementary Table S1**) were analyzed with unsupervised high-dimensional analysis, using a computational pipeline including the FlowSOM algorithm (36). The antigen-specific B cells were gated using combinatorial probe gating within live CD19+ B cells (**Supplementary Figure S2A**). In total, 30 different B cell clusters were identified when B cells of all six different antigen specificities were combined with bulk CD19+ B cells (**Figures 2B, C**). Overarching populations were identified based on expression of core B cell markers and subclusters were annotated based on presence of activation markers (**Figures 2C, D**). Similar populations were found previously by us upon deep profiling of specific B cell compartments in individuals having experienced COVID-19 with different grades of disease severity (28). Naïve B cells (IgD+, NBC), classical MBCs (CD27+CD38−) and PB (CD27+CD38+) populations were identified in the antigen-specific compartments. In addition, clusters were identified that contain characteristics of the atypical B cells, including Double Negative (CD11c+IgD-CD27−, DN) B cells, activated Naïve (CD11c+IgD+, aNAV) cells, and age-associated B cells (CD11c+CD27+, ABC), which we recently investigated and characterized after SARS-CoV-2 vaccination (39) and appear during the extrafollicular response prior to GC formation (40). Finally, IgA+, IgM+, and IgG+ activated B cell populations (ActBC) were identified being CD27+, CD71+, CD95+, and CD43+ (clusters 3, 4, and 5). Our previous research on B cell immune profiles after infection identified CD71 and CD43 as defining markers for these ActBC populations (28). Furthermore, a population of antigen-specific CD71+CD27+ B cells was previously shown to arise in the blood at the same time of GC formation after infection and vaccination (41). In addition, a population described as CD27 high but CD21 low B cells (42) shares similarities with this ActBC population and is shown to be early precursors prior to PB differentiation. Taken together, ActBC populations may reflect recent GC experience and be indicative of ongoing GC reactions. These data show that the used multiparameter approach can successfully identify specific B cell populations at different stages of extrafollicular and GC-driven differentiation, hereby generating in depth insight in the composition of antigen-specific B cell compartments and phenotypic B cell subset relationships after infection.

**Figure 3:**
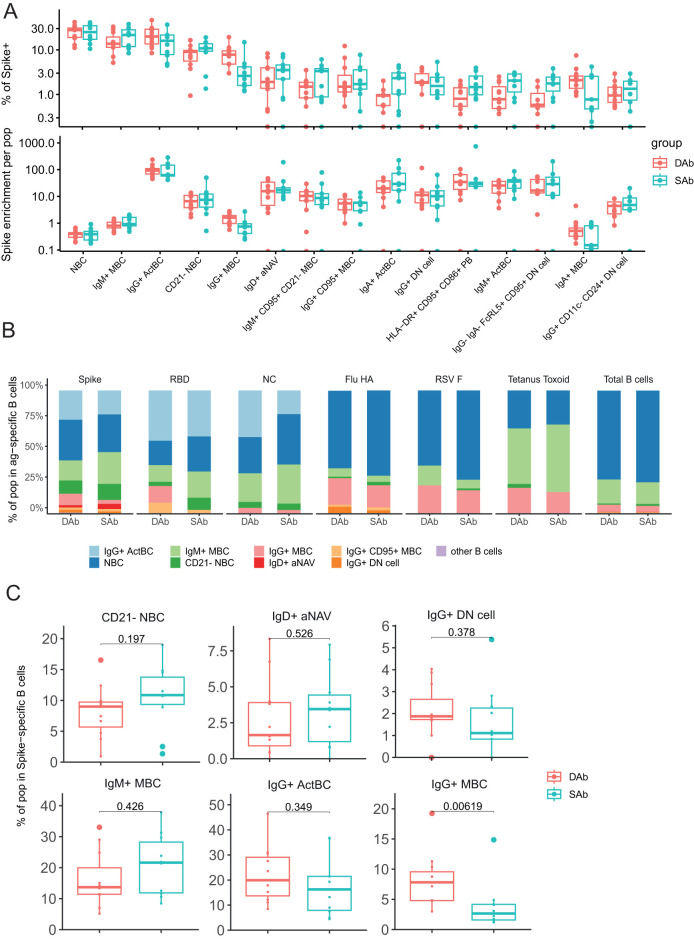
Decrease of SARS-CoV-2-specific IgG+ MBC in the sustained Ab group. **(A)** Frequency of B cell populations within Spike-specific B cells for DAb (n=11) and SAb (n=10) groups. Spike enrichment of each population was calculated by dividing the frequency in total CD19+ B cells by frequency of Spike-specific B cells. Order of populations was arranged by median frequency of Spike-specific populations. There were 15 most Spike-specific B cell populations that were plotted. **(B)** Relative frequency of eight frequent populations in Spike, RBD, and NC, and Flu HA-, RSV F-, and tetanus toxoid-specific B cells for DAb and SAb along with total B cells. **(C)** Comparison of frequency of selected populations of Spike-specific B cells for DAb and SAb. Mann–Whitney test was performed.

UMAP representations of the individual SARS-CoV-2-specific B cells in the SAb and DAb groups indicate that Spike-, RBD-, or NC-specific B cells (**Figure 2E**) show a different distribution across B cell clusters compared with Flu-, RSV-, or TT-specific B cells, in line with the more recent encounter of SARS-CoV-2 (**Supplementary Figure S2B**). To further investigate this, overall frequencies of the annotated B cell populations in Spike-specific B cells (**Figure 3A**), exceeding a preset minimal threshold of number of events (see M&M), were compared with those in total B cells (**Supplementary Figure S2C**), revealing the Spike-specific level of enrichment of annotated B cell populations (**Figure 3A**). The frequency of the eight most frequent populations in the Spike-specific compartment were next depicted for all antigens investigated and for total B cells (**Figure 3B**). IgG+ ActBC, as well as IgM+ and IgA+ ActBC, are clearly enriched in the Spike-specific B cell compartment of convalescent donors for both groups (**Figure 3A**). These populations are not present when studying total B cell compartments specific for Flu, RSV, or TT that were encountered longer ago (**Figure 3B**). In addition, IgG+ ActBC form a substantial fraction of the Spike-specific response (**Figure 3B**), indicating active ongoing GC reactions 6–8 weeks after mild infection. Also, HLA-DR+CD95+CD86+ PB are strongly enriched in both groups in the Spike-specific compartment. IgG+(CD95+) MBC and IgM+CD95+CD21− MBC (clusters 10, 11, and 14) are enriched for Spike specificity, but these populations are also observed in the B cell compartments of the TT, Flu, and RSV antigens encountered longer ago (**Figure 3B**), consistent with the formation of a long-lived memory response after infection. IgG+(CD11c-CD24+) DN cells are present in Spike-, RBD-, and Flu-specific B cells. CD21− NBC and aNAV (clusters 26 and 27) are among the most frequent Spike-specific B populations, which might indicate recently primed naïve populations (**Figures 3A, B**). Interestingly, CD21− NBC clustered together with the atypical phenotype in the UMAP and heatmap (**Figures 2B, C**) rather than other NBC. The majority of the Spike-specific B cell populations induced after SARS-CoV-2 are comparable in both DAb and SAb groups (**Figure 3C**). Strikingly, within the Spike- and RBD-specific B cells, the IgG+ MBC (**Figure 3C**, **Supplementary Figure S3A**) are a significantly higher proportion in the DAb group compared with the SAb group, with also a higher overall frequency of RBD-specific IgG+ MBCs (**Supplementary Figure S3B**). This indicates that people who generate stable SARS-SoV-2 antibody levels over time generated a relatively lower GC output toward a B cell memory phenotype at 6–8 weeks after mild infection compared with persons who show a greater decay of SARS-CoV-2-specific antibodies.

### Antibody neutralization capacity increased over time in the sustained antibody group

**Figure 4:**
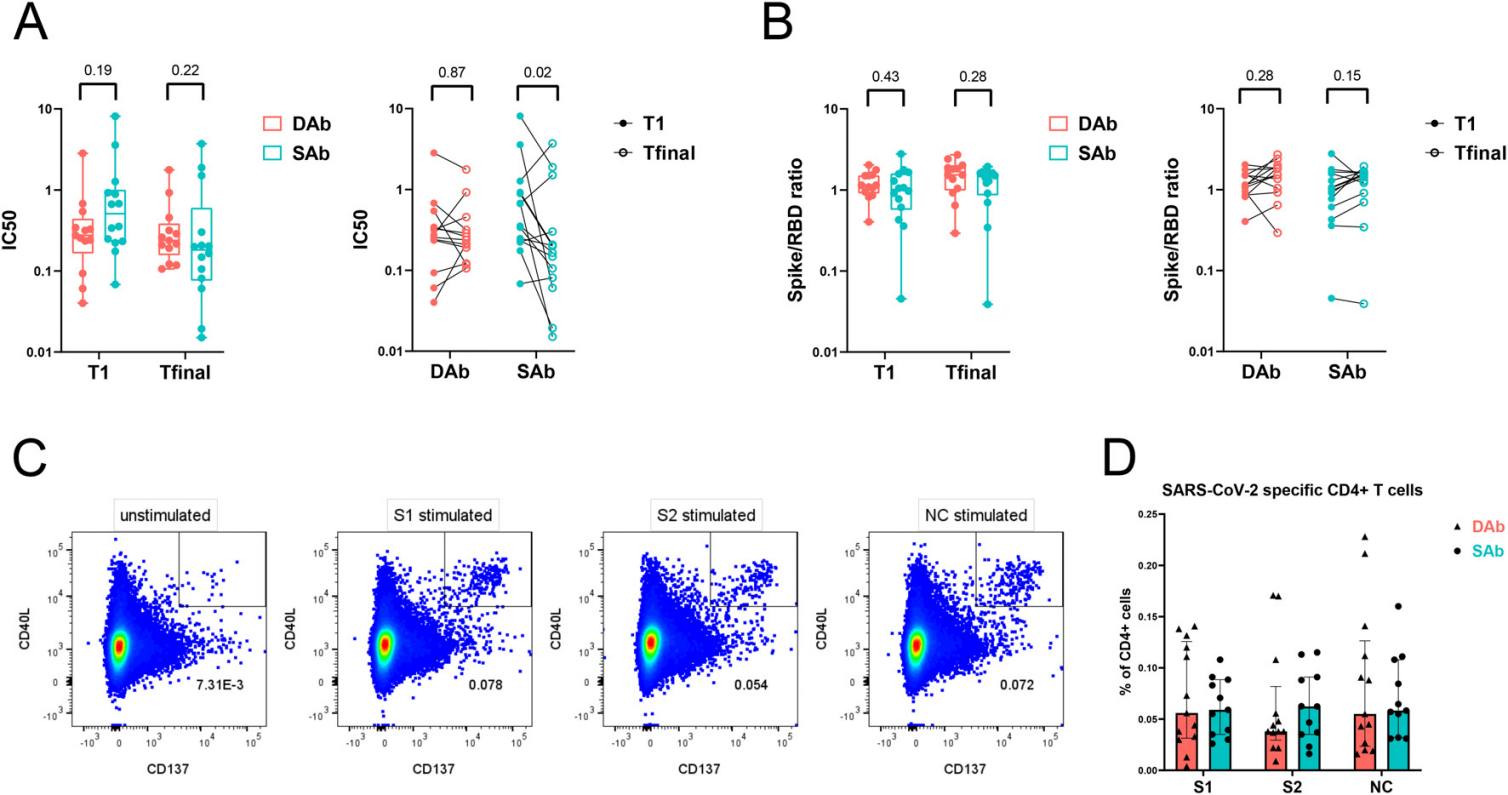
Neutralization potency of SARS-CoV-2 antibodies increase over time in SAb group whereas frequency of SARS-CoV-2-specific T cells is similar between the groups. **(A)** Neutralization capacity of RBD antibodies from plasma samples of DAb (n=13) and SAb (n=14) groups at time point of PBMC sampling (T1) and final measuring time point (Tfinal). **(B)** Ratio of Spike and RBD titers from plasma samples are displayed. Inter-time (ratio paired t-test) and inter-group (Mann–Whitney test) comparisons are shown. Box plot displays mean and extends from the 25th to 75th percentiles with whiskers to the smallest and largest value. **(C)** Representative flow cytometry plot of SARS-CoV-2-specific CD4+ T cells measured as percentage CD40L+ CD137+ CD4+ T cells after O/N stimulation of PBMCs with DMSO control (unstimulated), S1, S2, or NC peptide pool. **(D)** Frequency of SARS-CoV-2-specific CD4+ T cells after stimulation of PBMCs with peptide pools in the DAb (n=13) and SAb (n=11) convalescent donor groups. Data were background subtracted against DMSO negative control and are shown with median and interquartile range. Mann–Whitney test was performed.

Ongoing GC reactions are needed to generate antigen-specific antibodies with higher affinities due to the GC processes of somatic hypermutation and affinity maturation. Over time, this results in antibody outputs with enhanced neutralization capacities. Indeed, other studies have shown an increase in neutralization capacity of SARS-CoV-2 antibodies during late convalescence (16, 19). To study if the observed differences in B cell responses against SARS-CoV-2 between SAb and DAb groups are also reflected in the neutralization capacities of specific antibodies in both groups, the neutralizing potency of plasma samples was investigated at median 44 days (T1) and 128 days PSO (Tfinal) by a competitive ELISA assay. At both timepoints, there were no significant differences in neutralization potency between both groups. Interestingly, neutralizing potency increased significantly over a period of 5 months after infection in the SAb group, as demonstrated by a decrease in the IC50 value, whereas neutralization potency remained unchanged in the DAb group (**Figure 4A**). In vaccination studies, an antigenic shift toward less dominant and more conserved Spike epitopes after second and third vaccination has been described (43, 44). To examine if a broadening of the antibody repertoire to non-RBD epitopes occurred over time, the ratio of Spike to RBD antibody titers was studied and compared between the SAb and DAb groups at the two time points. The Spike to RBD ratio did not change significantly between T1 (median SAb: 1,01; DAb: 1,15) and Tfinal (median SAb: 1,46; DAb: 1,54) and was similar between both groups (**Figure 4B**). Together, these data show that individuals with stable antibody levels over time show an increase in neutralization capacity of the generated specific antibodies.

### No differences in SARS-CoV-2-specific T cells between sustained and declining antibody groups

As the SARS-CoV-2-specific immune response showed signs of GC reactions and thus T cell dependency, the SARS-CoV-2-specific CD4+ T cell response in circulation was compared between the SAb and DAb groups using an activation-induced marker assay. Upon stimulation with Spike (S1 or S2) or NC peptide pools, convalescent donors upregulated activation markers CD40L and CD137 compared with unstimulated control, revealing the SARS-CoV-2-specific CD4+ T cell population (**Figure 4C**, **Supplementary Figure S4A**). The frequency of SARS-CoV-2-specific CD4+ T cells at 45 days PSO was similar between the SAb and DAb groups, with a high inter-donor variability (**Figure 4D**). Phenotypic analysis of the SARS-CoV-2-specific CD4+ T cells displayed an enrichment of the central memory phenotype (CD27+ CD45RA−) compared with the whole CD4+ T cell population (**Supplementary Figure S4B**). The distribution and frequency of the various memory populations did not differ between the SAb and DAb groups (**Supplementary Figure S4C**). Characterization of the T helper phenotype using chemokine markers to identify a mixed population of T follicular and T helper (T (follicular) helper) 17 cells (T(f)h17, CCR6+), T (follicular) helper 1 cells (T(f)h1, CXCR3+, CCR6−), and T (follicular) helper 2 cells (T(f)h2, CCR4+, CXCR3−, CCR6−) demonstrated a significant enrichment and dominance of T(f)h1 cells in the SARS-CoV-2-specific population compared with total non-naïve CD4+ T cells, and a lower frequency of T(f)h17 and T(f)h2 cells was observed in both the SAb and DAb groups (**Supplementary Figure S5A**). No significant differences in the percentage of the T helper cell populations in SARS-CoV-2-specific CD4+ T cells were found between the SAb and DAb groups (**Supplementary Figure S5B**), indicating that early after mild infection, the balance between T(f)h1, T(f)h2, and T(f)h17 cells is not associated with longevity of the SARS-CoV-2 antibody response. In support, frequencies of SARS-CoV-2-specific cytokine producing CD4+ T cells did not differ between groups, also not for IL-21 producing CD4+ T cells (**Supplementary Figure S6**).

### Spike-specific B cell populations correlated with Spike-specific CD4+ T cells to a greater extent in the sustained antibody group

**Figure 5:**
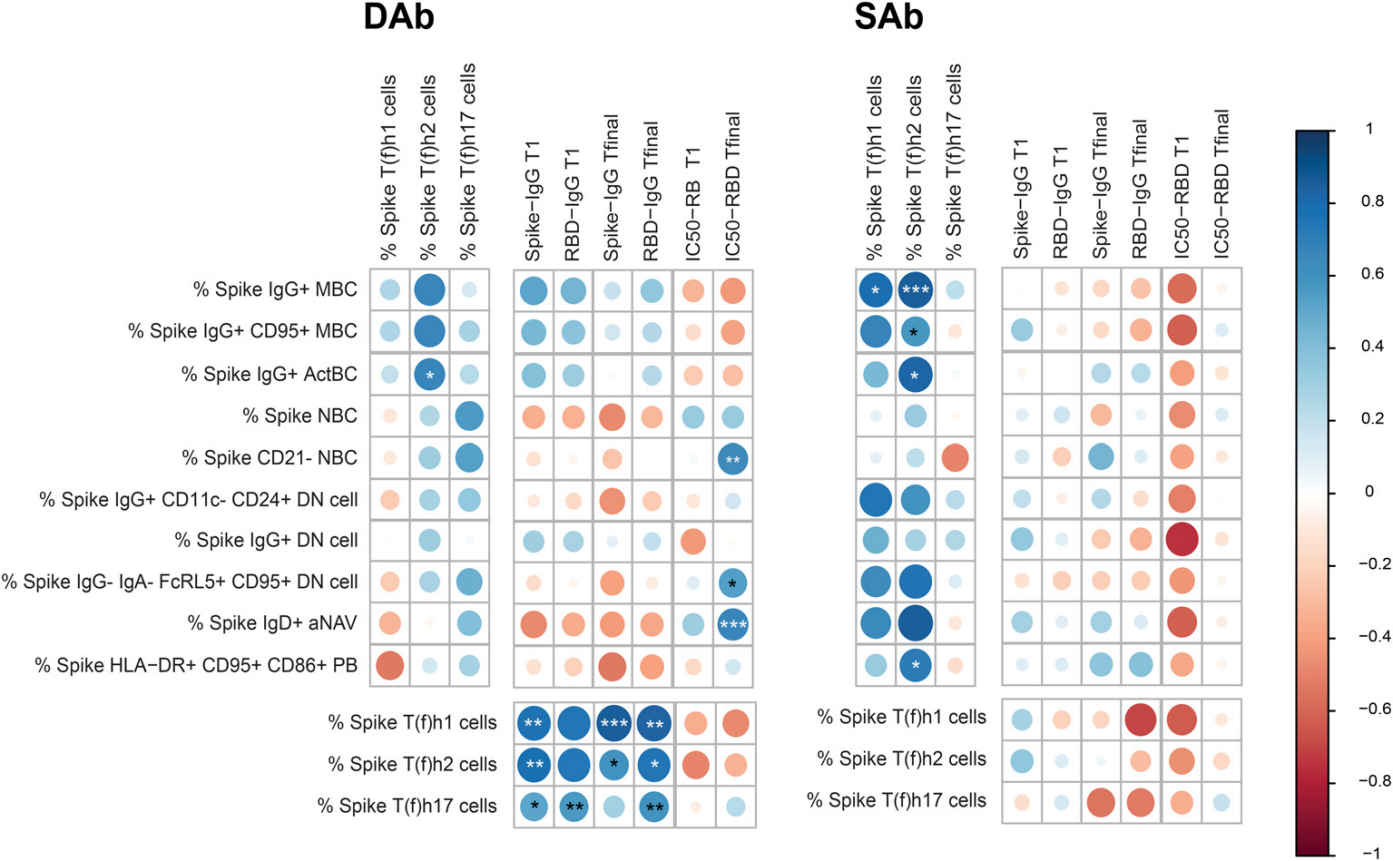
Correlations between SARS-CoV-2-specific B cell, T cell, serology, and neutralization capacity in the DAb and SAb groups. Correlation matrix between Spike-specific B cell populations as frequency of CD19+ B cells with Spike-specific T-cell populations as frequency of CD4+ T cells and Spike titers and neutralization capacity of RBD Ab at T1 and Tfinal. The correlations were performed with the corrplot package to visualize the data. Spearman correlations were performed to analyze the data. The color of the circle indicates a positive or negative correlation, with the asterisks as indicators of statistical significance. The size of the circles and intensity of the colors show the value of the corresponding correlation coefficients. For the IC50, a lower value indicates a stronger neutralization capacity, so a positive correlation indicates an inverse association with neutralizing ability. Number of donors included for each set of correlations: B-T correlations included 18 donors (11 DAb and 7 SAb), B-Ab correlations 21 donors included (11 DAb and 10 SAb) and T-Ab correlations included 24 donors (13 DAb and 11 SAb). *P < 0.05, **P < 0.01, ***P < 0.001.

To assess if and how cellular and humoral parameters relate in both SAb and DAb groups, circulating SARS-CoV-2-specific B and T cell subset frequencies, antibody titers, and neutralization capacity were correlated in both SAb and DAb groups. Clear differences were observed in the overall pattern of correlations between Spike-specific B and T cell subpopulations and antibody titers between DAb and SAb groups (**Figure 5**). For the DAb group, Spike-specific IgG+ ActBC cell populations positively correlate with the Spike-specific T(f)h2 cell populations (**Figure 5**, **Supplementary Figure S7A**). In comparison, in the SAb group, not only did IgG+ ActBC and MBC B cell populations strongly correlate with Spike-specific T (f)h2 (**Supplementary Figure S7A**) but also additional correlations were observed between distinct atypical B cell and PB populations with T(f)h1 and T(h)2. In support, Spike-specific IgG+ MBC also strongly correlated with Spike-specific IFNγ+ or TNF+ CD4+ T cells, whereas some Spike-specific IgG+ DN cells correlated with Spike-specific IL-21 producing CD4+ T cells (**Supplementary Figure S7B**). None of the B cell populations correlated with antibody titers at T1 or Tfinal for either group. For the SAb group, the Spike-specific B cell subsets had a tendency toward positive correlation with the neutralization capacity at T1 in contrast to the DAb group; however, the DAb group displayed a strong negative correlation with the IC50 at the final timepoint for several B cell subsets. All Spike-specific CD4+ T cell helper subsets including cytokine-producing T cells were strongly correlated in the DAb groups with antibody titers at both timepoints, which was not the case for the SAb (**Figure 5**, **Supplementary Figures S7A, B**). In conclusion, the DAb group showed strong correlations between Spike-specific CD4+ T cells and Spike-specific IgG antibodies, but overall no major correlations between B cells with CD4+ T cell or antibodies was found. In contrast for the SAb group, a larger array of correlations between Spike-specific B cell populations and CD4+ T cells were observed but no cellular correlations with Spike-IgG titers.

## Discussion

For many years, it is a known phenomenon that maintenance of specific antibody levels after antigen encounter may differ between individuals. Persons that mount an especially long-lived antibody response are sometimes referred to as “elite” responders (29). Indeed, COVID-19 convalescent individuals with sustained SARS-CoV-2-specific IgG production have been described (30, 45–47) and were found to be correlated with rapid recovery (45). These studies showed that these donors are characterized by increased early somatic hypermutation and increased SARS-CoV-2-specific memory Tfh. Deep B cell profiling data comparing persons with sustained and declining antibodies has not been performed yet. Elucidation of differences in the B cell response in sustained responders versus fast antibody decliners will help to identify the mechanisms controlling longevity of specific antibody responses. Therefore, we made use of a cohort of convalescent donors consisting of 132 individuals who had mild disease and were followed over 5 months in the early stages of the SARS-CoV-2 pandemic. Individuals showed a heterogeneous response with mostly declining antibody titers over time. Some individuals, however, maintained stable antibody levels over the follow-up period. Linear mixed-effects modeling integrating all antibody measurements in time was used to group the individuals. Individuals who showed the most rapidly declining or most sustained anti-RBD IgG titers of the cohort were analyzed for circulating SARS-CoV-2-specific B and T cell immunity and compared. As there were no clear differences in half-life for NC antibodies between the two groups, we focused on the Spike- and RBD-specific responses.

In general, analysis of Spike/RBD-specific B cell subsets around 40 days PSO showed clear enrichment of (IgG+) ActBC, indicative of ongoing GC responses in all donors 6–8 weeks after mild infection. We were able to detect some PB clusters, although there were no significant differences detected between the groups. In this study, we did not to further focus on the PB/PC compartment since PB and PC are vulnerable to freeze-thawing (to which samples were subjected) leading to a potential underestimation of PBs and PCs or subsets of those lineages. Performing ELIspot would have been a good approach to investigate the PB and PC in more detail; however, freeze-thawing of the samples would also have generated an underestimation using this method. The appearance of IgG+(CD95+) MBC and IgM+CD95+CD21− MBC populations demonstrated formation of memory cells, which in the case of the IgG+ MBC compartment was significantly lower in the sustained group. One possible explanation for this result may lie in different dynamics of PC versus MBC formation for donors with sustained antibodies at this time point. This possibility may have been further explored in longitudinal samples. As sampling of our cohort did not allow this, future investigation is warranted to answer this. Another option for the differences here observed in peripheral blood may lie in the possibility that in the two groups, MBCs localize differently in the human body through differences in induction or retention and/or receptors. More studies, including aspirates from lymph nodes at multiple time points after antigen encounter, might give more insights. A third underlying explanation may be inherent differences in the output of the GC reactions between persons yielding sustained and declining antibody responses. It may be that after infection in donors with sustained antibodies, GC reactions are more prone to generate long-lived PC equipped to home to the bone marrow. Our observation that differences exist in the IgG+ MBCs early after infection indeed points to intrinsically different GC responses between both groups.

In addition, enrichment of aNAV, CD21- NBC, and DN was observed in the SARS-CoV-2-specific B cell populations. Enrichment of the aNAV population is in line with our previous data showing enrichment of aNAV B cells in the Spike-specific compartment upon SARS-CoV-2 vaccination (39). CD21− B cells can be increased in chronic viral infections and autoimmune diseases (48–52), and it is assumed that this expansion occurs due to chronic activation by the antigen. However, CD21− B cells are a mix of both memory and naïve phenotype and may be distinguished based on a combination of other activation markers such as CD38, CD24, CD95, CD11c, and FCRL4. The CD21− B cells described here cluster with naïve cells based on IgD expression. Absence of CD21 is a known characteristic for atypical B cells (DN B cells), and indeed the CD21− B cells in our data are in close phenotypic relationship to the aNAV, which also belong to the atypical B cells. The observed enrichment of these clusters in the SARS-CoV-2-specific B cell compartment may point to activation of the extrafollicular B cell differentiation pathway, in line with previous findings (53). Together, our data on B cell differentiation in convalescent donors after mild disease points to ongoing GC reactions around 6–8 weeks PSO with added contribution of extrafollicular responses. These dynamics are in line with previous reports of others and ourselves on SARS-CoV-2-specific B cell responses after COVID-19 (11, 28, 53), thus allowing us to use our deep B cell profiling approach for comparison between donors with different rates of Spike-specific antibody decay.

Upon comparison of donors with sustained and declining antibody responses, we did not observe differences in the overall frequency of SARS-CoV-2-specific T and B cells between groups. In-depth immunophenotyping of Spike/RBD-specific B cells, however, showed an enhanced proportion of IgG+ memory B cells in the DAb group early after infection. This indicates that at 6–8 weeks PSO, the output of the GC response in donors with sustained antibodies was clearly different from donors with declining antibodies, with the latter group already being in the process of generating an MBC output. In line with the different GC dynamics, we elucidated that donors with sustained antibodies also showed an increased neutralization capacity of their specific antibodies in time. These data suggest that the GC dynamics of “sustainers” are indeed different and longer maintained.

A few studies compared antigen-specific antibodies, B cells, and CD4+ T cells in the same SARS-CoV-2 convalescent individuals (54, 55). However, deep profiling to the extent that was performed in this study has not been done before. Consequently, correlations between SARS-CoV-2 specific B and T subsets have not been shown to this extent. In the SAb group, both the Spike-specific IgG+ ActBC and MBC correlate more strongly with Spike-specific T(f)h2 and T(f)h1 responses than in the DAb group. In addition, compared with the DAb group, overall more positive correlations were observed between the various Spike-specific B cell subsets and the T(f)h cell compartments in the SAb group. These positive correlations between specific B and Th cells may point to mutual feedforward communication in specific B and Th cell expansion in ongoing GC reactions specifically in the SAb group at the time point of analysis, in line with the GC-experienced nature of IgG+ ActBC and MBC. In contrast, in the DAb group, a stronger correlation is observed between Spike-specific T(f)h1 and T(f)h2 subsets and Spike and RBD-IgG titers at T1 and Tfinal timepoints. This may hint that the T(f)h cells form a limiting factor in both formation and maintenance of specific antibodies in persons with a declining antibody profile, but not in persons who generate stable antibodies over time. A potential explanation for the differential role of T(f)h cells observed might be distinct contribution of CXCR5+ Tfh cells, which could not be distinguished in this study. Increased SARS-CoV-2-specific memory Tfh cells were reported in COVID-19 convalescent individuals exhibiting sustained antibody titers (46). Previous studies of SARS-CoV-2-infected patients, additionally, showed that CXCR3+ CCR6− Tfh1 cells correlated positively with titers of neutralizing antibody indicating a role of these cells in antibody maintenance and viral clearance in COVID-19 (56, 57). In a mouse model of acute Zika virus infection, Tfh1 cells were shown to be responsible for isotype class switching of the IgG2a or IgG2c antibodies and essential for induction and long-term maintenance of protective neutralizing antibodies (58).

Although we come closer to uncovering the differences in the B cell compartment between persons with sustained and declining antibodies, the underlying mechanisms leading to the generation of sustained antibody levels after SARS-CoV-2 exposure in some individuals and not in others remain to be elucidated. Future investigations are needed to first validate our findings and second to elucidate the dynamics of the B cell responses in persons with sustained and declining antibody responses at multiple time points after infection. Our finding that specifically donors with sustained antibodies showed increased neutralization capacity of their antibodies over time makes it likely that their GC responses are more long-lasting than in donors with declining antibodies. Potentially better and/or more long-term retention of antigens may play a role (16, 59). The fact that the convalescent donors analyzed here all demonstrated mild infection argues against an exceptionally high or long-lasting viral load as a key factor for higher or more prolonged antigen exposure. It is possible that in “sustainers,” antigen is captured better or longer by the follicular dendritic cells (FDC) network in GC, thus driving different and prolonged GC responses. In this respect, the recent finding that in mice central FDCs are responsible for long-term antigen-immune complex retention in a complement receptor type 2 (CR2)-dependent manner may make it worth exploring if genetic variation in CR2 or other factors of the complement system forms a discriminating factor for “sustainers” (60). However, it should be noted that we and others do not find a difference in neutralization capacity between sustainers and decayers (30, 47). Future more extensive longitudinal studies are needed to elaborate on our findings and relate interpersonal differences in maintenance of *de novo* antibody levels, e.g., after vaccination for new antigens such as Rabies, with development of ASC and MBC populations over time. In the ideal scenario, samples would be obtained from lymph nodes and bone marrow to allow a more in-depth study of the GC response and to investigate the differences in maintenance of PC compartment.

In summary, we show that there are differences in the T cell-driven B cell response and GC reactions between donors with sustained or declining specific antibody levels after SARS-CoV-2 infection. It is important to understand the mechanisms behind the heterogeneity of these responses as the longevity of antibodies raised against pathogens or vaccines forms an important factor to maintain protection against subsequent infection. In addition, insights in key factors that control formation of long-lasting antibody responses may be used to prevent undesired, sustained antibody responses upon transplantation or in autoimmunity.

## Data Availability

All data is readily available in the main text and **Supplementary Materials**. Flow Cytometry Standard data generated in this study will be deposited at Zenodo (B cell data at https://doi.org/10.5281/zenodo.14198396 and T cell data at https://doi.org/10.5281/zenodo.14217236).

## Supplementary material

Supplementary Figure 1 Additional information on cohort of convalescent individuals with declining or sustained RBD-IgG antibody titers. **(A)** Overview of RBD-IgG half-life for all 132 donors included in this study. **(B)** Anti-Spike IgG titers measured up to 157 days PSO for DAb (n=13) and SAb (n=14) groups. **(C)** Spike IgG half-life of DAb and SAb donors, as estimated using linear mixed-effects modeling integrating all measuring points. In case of rising antibody levels, half-life in days was arbitrarily assigned a value of 4000. Individual values and median are shown. **(D)** Anti-NC IgG titers measured up to 157 days PSO for the DAb and SAb group. **(E)** NC IgG half-life of DAb and SAb donors, as estimated using linear mixed-effects modeling integrating all measuring points. Individual values and median are shown.

Supplementary Figure 2 Differences in B cell populations between DAb and Sab. **(A)** Gating strategy for live CD19+ B cells and antigen-specific cells before analysis using R. RBD-specific B cells are gated from the Spike+ gate. **(B)** UMAP distribution of equal number of antigen-specific cells in the DAb (n=13) and SAb (n=14) cohorts for Influenza, RSV and TT antigens. **(C)** Frequency of B cell populations in total CD19+ B cells for DAb and SAb groups.

Supplementary Figure 3 Comparison in Spike- and RBD-specific B cells between DAb and Sab. **(A)** Comparison of frequency of other analyzed populations within Spike-specific B cells for DAb (n=13) and SAb (n=14) groups. **(B)** Comparison of frequency of all selected populations within RBD-specific B cells for DAb and SAb. Mann-Whitney test was performed.

Supplementary Figure 4 Characterization of SARS-CoV-2 specific T cell immune response in DAb and SAb group. **(A)** Gating strategy shown to identify SARS-CoV-2-specific CD4+ T cells measured as percentage CD40L+ CD137+ CD4+ T cells and further phenotype naïve/memory and T helper cell populations. **(B)** Distribution of naïve and memory populations in SARS-CoV-2-specific CD4+ T cells in the DAb (n=13) and SAb (n=14) group stimulated with S1, S2 or NC peptide pool compared to total non-naïve CD4+ T cells. Data is shown with geometric mean and geometric standard deviation. An unpaired t-test with Welch correction and multiple comparison correction according to Holm-Šídák was performed. **(C)** Frequency of SARS-CoV-2-specific Tem, Tcm, naïve and Temra cells after stimulation of PBMCs with S1, S2 and NC peptide pool in the DAb and SAb convalescent donor group. Data was background subtracted against DMSO negative control and are shown with median and interquartile range. Mann-Whitney test was performed.

Supplementary Figure 5 T helper phenotype of SARS-CoV-2 specific T cells in DAb and SAb group. **(A)** Distribution of T helper populations in SARS-CoV-2-specific CD4+ T cells in the DAb (n=13) and SAb (n=14) group stimulated with S1, S2 or NC peptide pool compared to total non-naïve CD4+ T cells. Data is shown with geometric mean and geometric standard deviation. An unpaired t-test with Welch correction and multiple comparison correction according to Holm-Šídák was performed. **(B)** Frequency of SARS-CoV-2-specific T(f)h1, T(f)h2 and T(f)h17 cells after stimulation of PBMCs with S1, S2 and NC peptide pool in the DAb and SAb convalescent donor group. Data was background subtracted against DMSO negative control and are shown with median and interquartile range. Mann-Whitney test was performed.

Supplementary Figure 6 Cytokine producing SARS-CoV-2 specific T cells in DAb and SAb group. Frequency of SARS-CoV-2-specific IL21+ **(A)**, IFNγ+ TNF+ **(B)**, TNF+ **(C)** and IFNγ+ **(D)** CD4+ T cells after stimulation of PBMCs with S1, S2 and NC peptide pool in the DAb (n=13) and SAb (n=14) convalescent donor group. Data were background subtracted against DMSO negative control and are shown with median and interquartile range. Mann-Whitney test was performed.

Supplementary Figure 7 Correlations between B cell and T helper subsets. **(A)** Spearman correlations between certain B cell and T cell subsets showing the correlation line and significance. **(B)** Correlation matrix between Spike-specific B cell populations as frequency of CD19+ B cells with Spike-specific cytokine-producing T cell populations as frequency of CD4+ T cells and Spike titers and neutralization capacity of RBD Ab at T1 and Tfinal.

Supplementary Table 1 Information on antibodies and probe fluorophores used in the spectral flow cytometry analysis of B cells.

Supplementary Table 2 Information on probe fluorophores used in the spectral flow cytometry analysis of B cells.

Supplementary Table 3 Information on antibodies used in the AIM assay to detect and characterize SARS-CoV-2-specific T cells.
