# Supplementary material for: Longevity of antibody responses is associated with distinct antigen-specific B cell subsets early after infection
Source: Front Immunol. 2024 Dec 17;15:1505719. doi: 10.3389/fimmu.2024.1505719 (PMC11686410; doi:10.3389/fimmu.2024.1505719)
Supplement: Supplementary Figure 1 — Additional information on cohort of convalescent individuals with declining or sustained RBD-IgG antibody titers. (A) Overview of RBD-IgG half-life for all 132 donors included in this study. (B) Anti-Spike IgG titers measured up to 157 days PSO for DAb (n=13) and SAb (n=14) groups. (C) Spike IgG half-life of DAb and SAb donors, as estimated using linear mixed-effects modeling integrating all measuring points. In case of rising antibody levels, half-life in days was arbitrarily assigned a value of 4000. Individual values and median are shown. (D) Anti-NC IgG titers measured up to 157 days PSO for the DAb and SAb group. (E) NC IgG half-life of DAb and SAb donors, as estimated using linear mixed-effects modeling integrating all measuring points. Individual values and median are shown. [file DataSheet1.pdf]

Figure S1

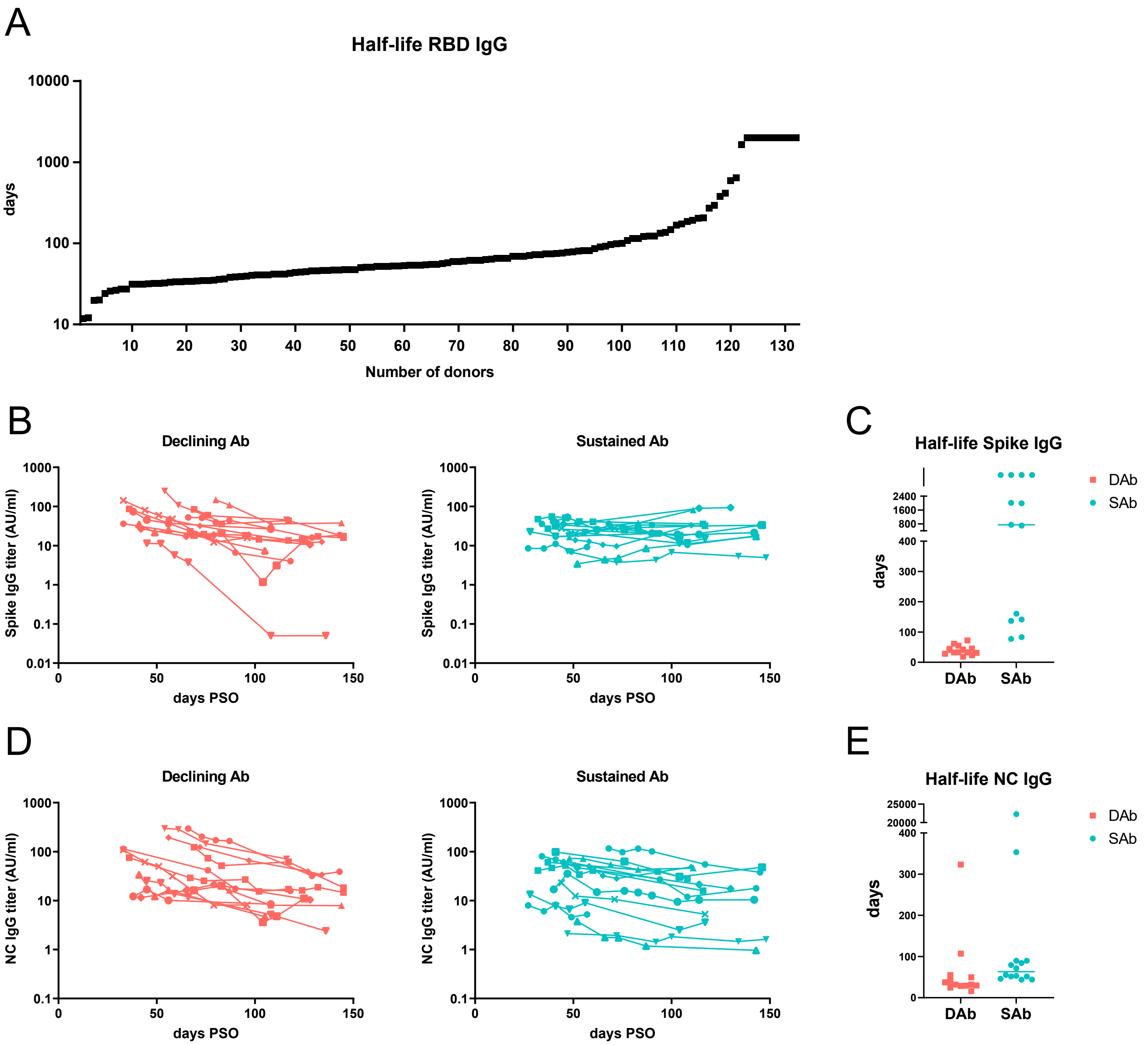
A
Half-life RBD IgG
days
10000
1000
100
10
10 20 30 40 50 60 70 80 90 100 110 120 130
Number of donors
B
Declining Ab
Sustained Ab
Spike IgG titer (AU/ml)
1000
100
10
1
0.1
0.01
0 50 100 150
days PSO
C
Half-life Spike IgG
2400
1600
800
400
300
200
100
0
days
DAb
SAb
D
Declining Ab
Sustained Ab
NC IgG titer (AU/ml)
1000
100
10
1
0.1
0 50 100 150
days PSO
E
Half-life NC IgG
25000
20000
400
300
200
100
0
days
DAb
SAb

Figure S2

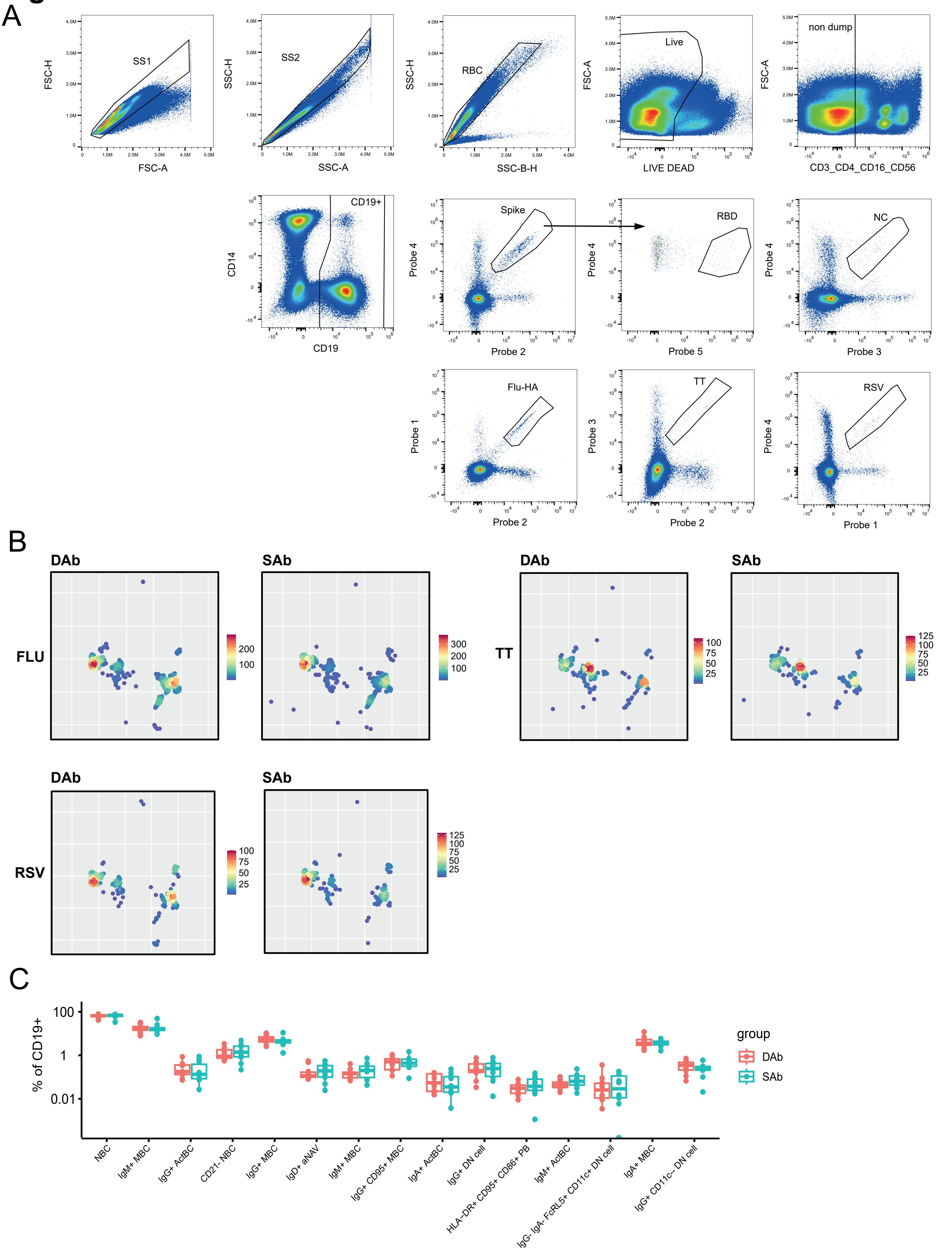
A
SS1
FSC-H
FSC-A
SS2
SSC-H
SSC-A
RBC
SSC-H
SSC-B-H
Live
FSC-A
LIVE DEAD
non dump
FSC-A
CD3_CD4_CD16_CD56
CD19+
CD14
CD19
Spike
Probe 4
Probe 2
RBD
Probe 4
Probe 5
NC
Probe 4
Probe 3
Flu-HA
Probe 1
Probe 2
TT
Probe 3
Probe 2
RSV
Probe 4
Probe 1
B
DAb
SAb
FLU
TT
RSV
C
% of CD19+
group
DAb
SAb
NBC
IgM+ MBC
IgG+ ActBC
CD21- NBC
IgG+ MBC
IgD+ aNAV
IgM+ MBC
IgG+ CD95+ MBC
IgA+ ActBC
IgG+ DN cell
HLA-DR+ CD95+ CD86+ PB
IgM+ ActBC
IgG- IgA- FcRL5+ CD11c+ DN cell
IgA+ MBC
IgG+ CD11c– DN cell

Figure S3

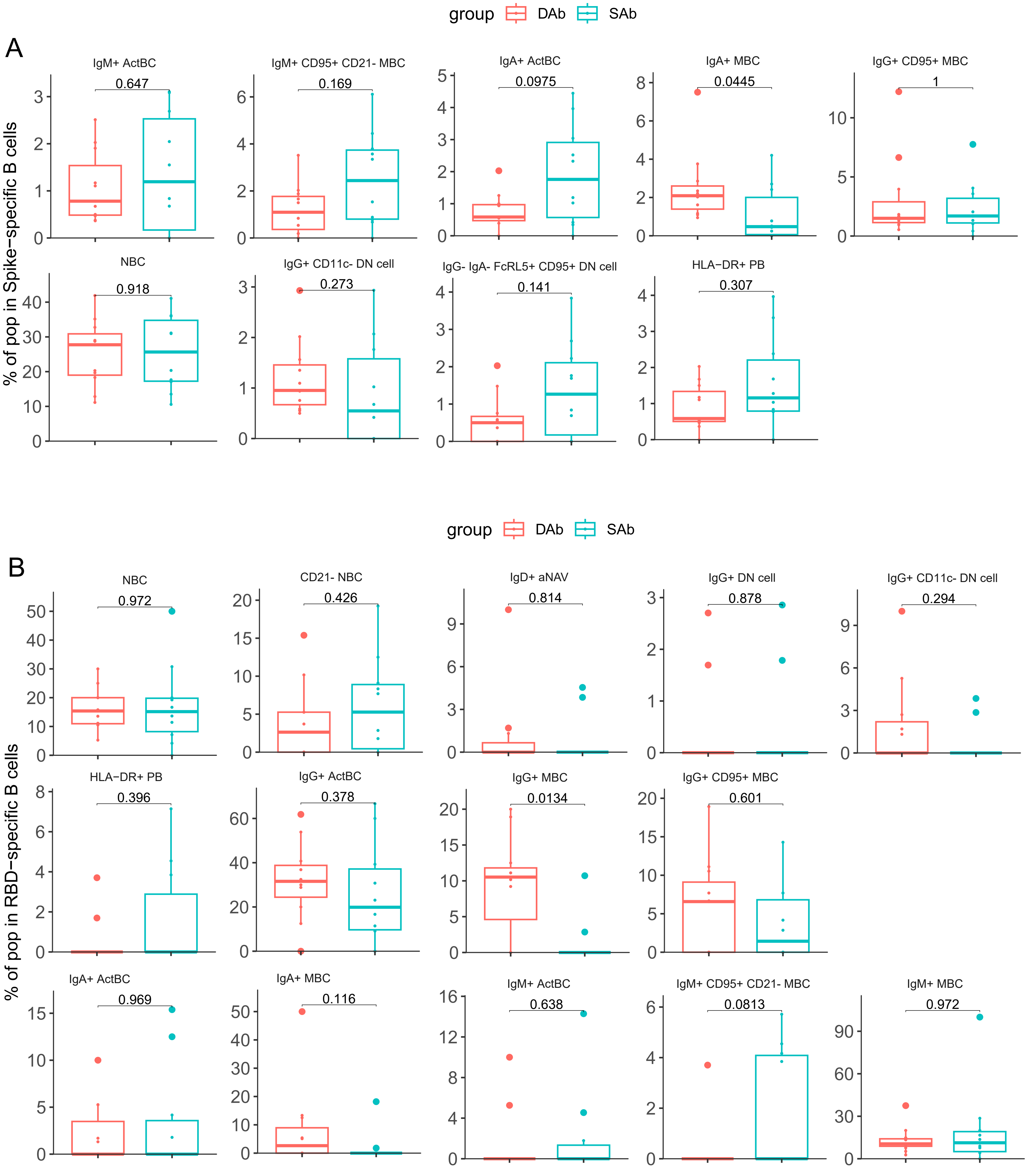
group DAb SAb
A
% of pop in Spike−specific B cells
IgM+ ActBC
0.647
IgM+ CD95+ CD21- MBC
0.169
IgA+ ActBC
0.0975
IgA+ MBC
0.0445
IgG+ CD95+ MBC
1
NBC
0.918
IgG+ CD11c- DN cell
0.273
IgG- IgA- FcRL5+ CD95+ DN cell
0.141
HLA−DR+ PB
0.307
group DAb SAb
B
% of pop in RBD−specific B cells
NBC
0.972
CD21- NBC
0.426
IgD+ aNAV
0.814
IgG+ DN cell
0.878
IgG+ CD11c- DN cell
0.294
HLA−DR+ PB
0.396
IgG+ ActBC
0.378
IgG+ MBC
0.0134
IgG+ CD95+ MBC
0.601
IgA+ ActBC
0.969
IgA+ MBC
0.116
IgM+ ActBC
0.638
IgM+ CD95+ CD21- MBC
0.0813
IgM+ MBC
0.972

Figure S4

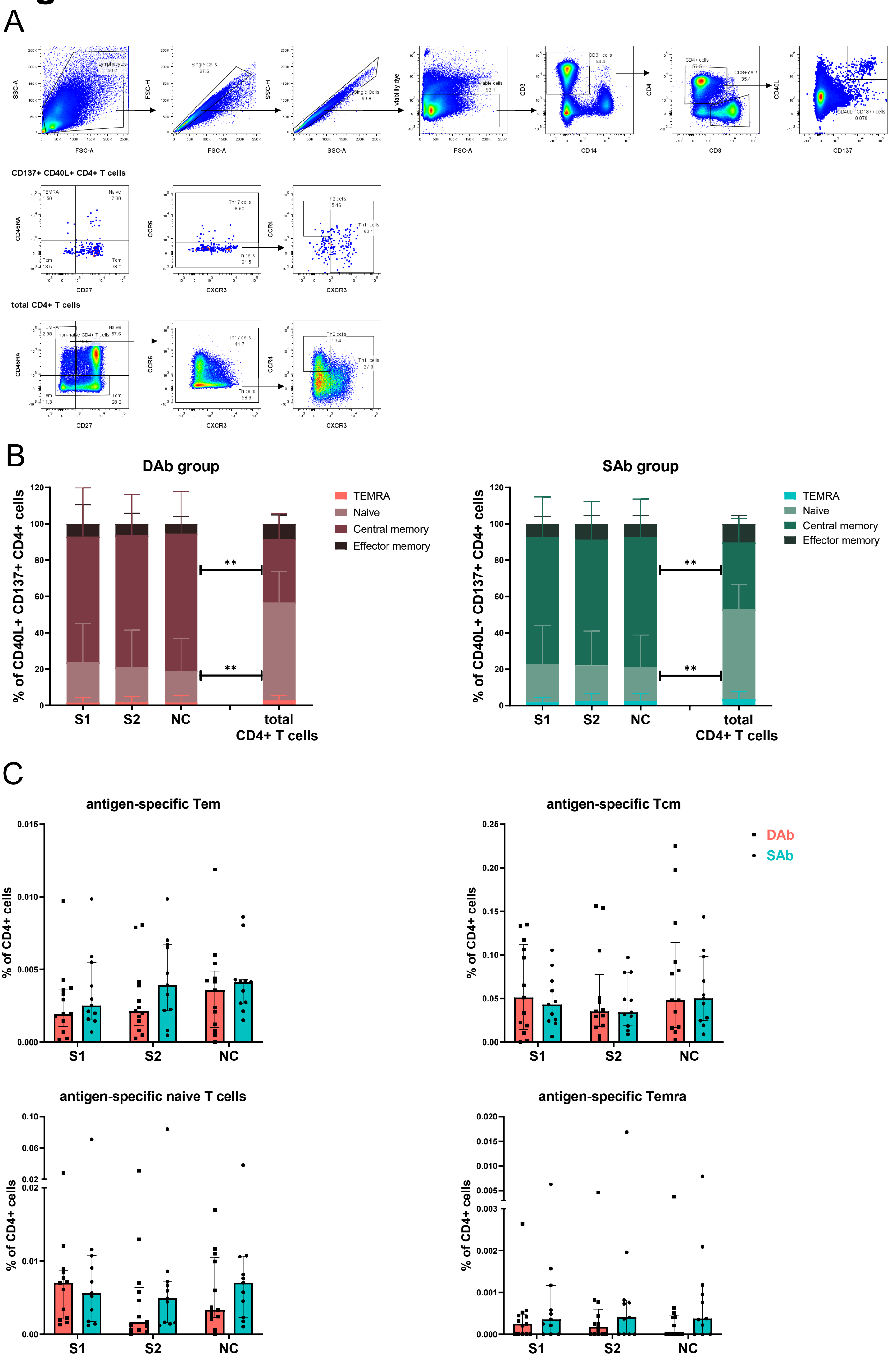
A
SSC-A
FSC-A
Lymphocytes 58.2
FSC-H
Single Cells 97.6
SSC-H
SSC-A
Single Cells 99.8
viability dye
viable cells 92.1
CD3
CD14
CD3+ cells 54.4
CD4
CD8
CD4+ cells 57.5
CD8+ cells 35.4
CD40L
CD137
CD40L+ CD137+ cells 0.078
CD137+ CD40L+ CD4+ T cells
CD45RA
CD27
TEMRA 1.50
Naive 7.00
Tem 13.5
Tcm 78.0
CCR6
CXCR3
Th17 cells 8.50
Th cells 91.5
CCR4
Th2 cells 5.46
Th1 cells 60.1
total CD4+ T cells
TEMRA 2.98
Naive 57.6
non-naive CD4+ T cells 43.0
Tem 11.3
Tcm 28.2
Th17 cells 41.7
Th cells 58.3
Th2 cells 19.4
Th1 cells 27.0
B
DAb group
SAb group
% of CD40L+ CD137+ CD4+ cells
TEMRA
Naive
Central memory
Effector memory
**
S1
S2
NC
total CD4+ T cells
C
antigen-specific Tem
antigen-specific Tcm
antigen-specific naive T cells
antigen-specific Temra
% of CD4+ cells
DAb
SAb

Figure S5

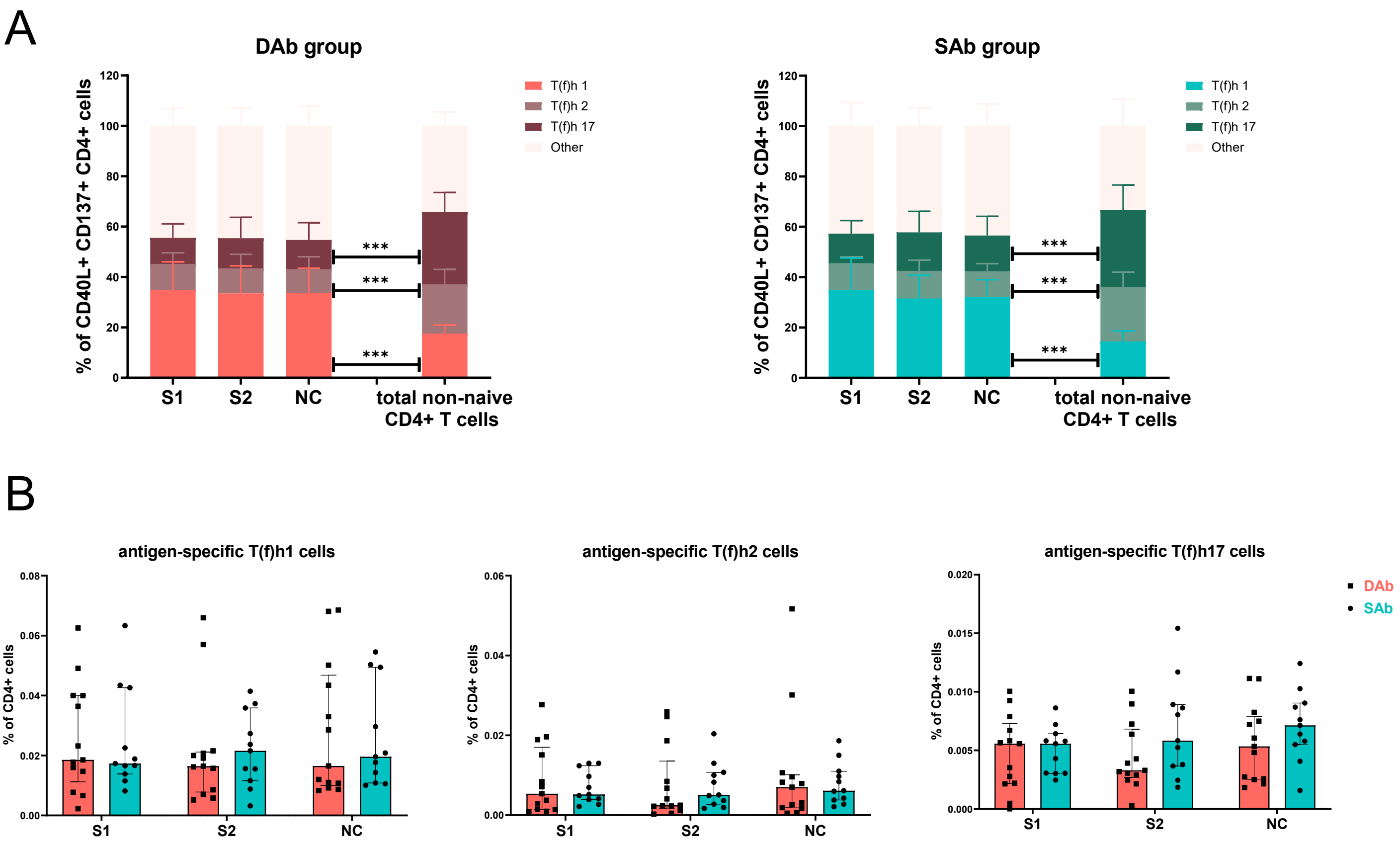
A
DAb group
% of CD40L+ CD137+ CD4+ cells
T(f)h 1
T(f)h 2
T(f)h 17
Other
***
***
***
S1
S2
NC
total non-naive CD4+ T cells
SAb group
B
antigen-specific T(f)h1 cells
antigen-specific T(f)h2 cells
antigen-specific T(f)h17 cells
% of CD4+ cells
DAb
SAb

Figure S6

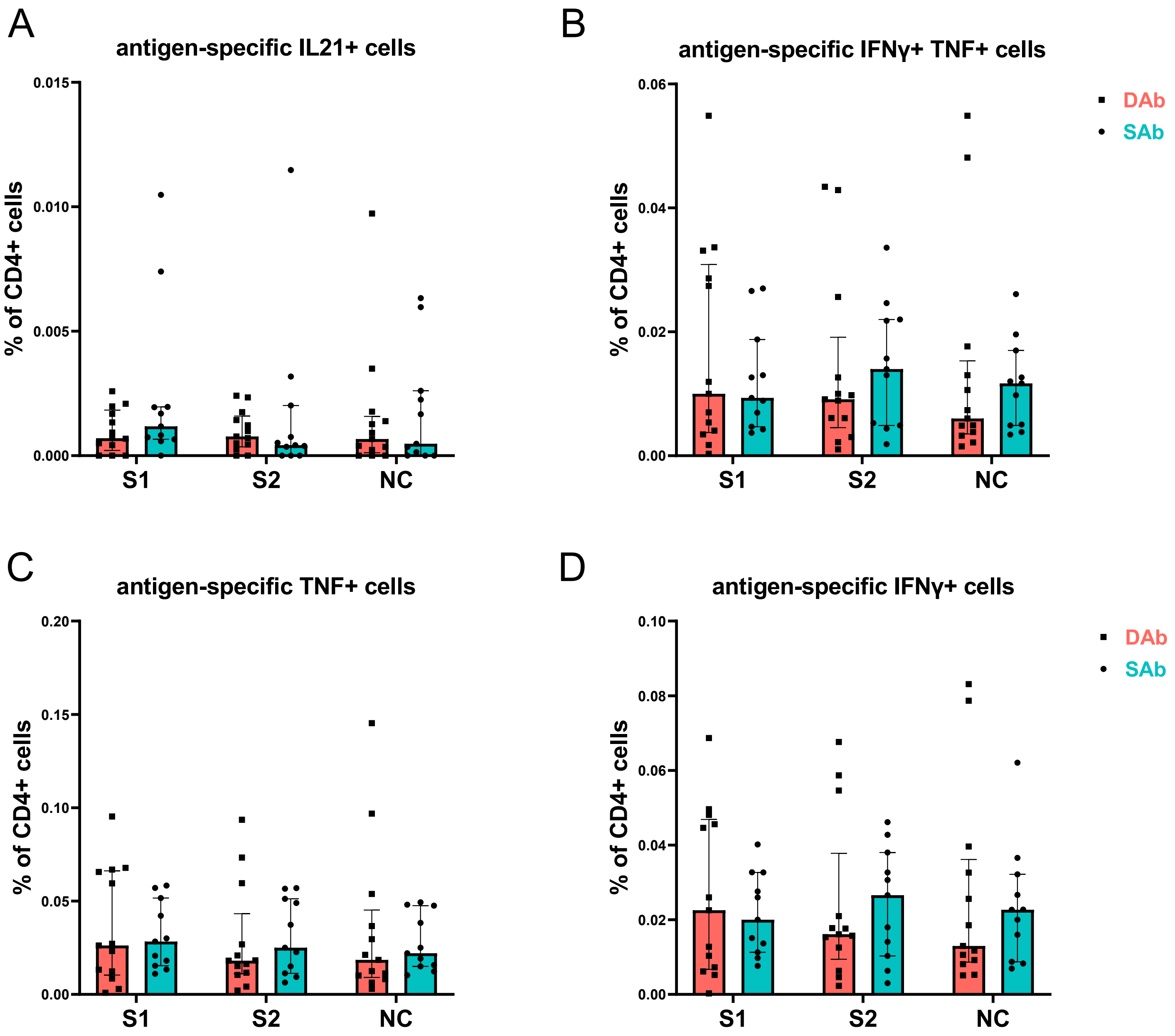
A
antigen-specific IL21+ cells
% of CD4+ cells
0.015
0.010
0.005
0.000
S1
S2
NC
B
antigen-specific IFNγ+ TNF+ cells
% of CD4+ cells
0.06
0.04
0.02
0.00
S1
S2
NC
DAb
SAb
C
antigen-specific TNF+ cells
% of CD4+ cells
0.20
0.15
0.10
0.05
0.00
S1
S2
NC
D
antigen-specific IFNγ+ cells
% of CD4+ cells
0.10
0.08
0.06
0.04
0.02
0.00
S1
S2
NC
DAb
SAb

Figure S7

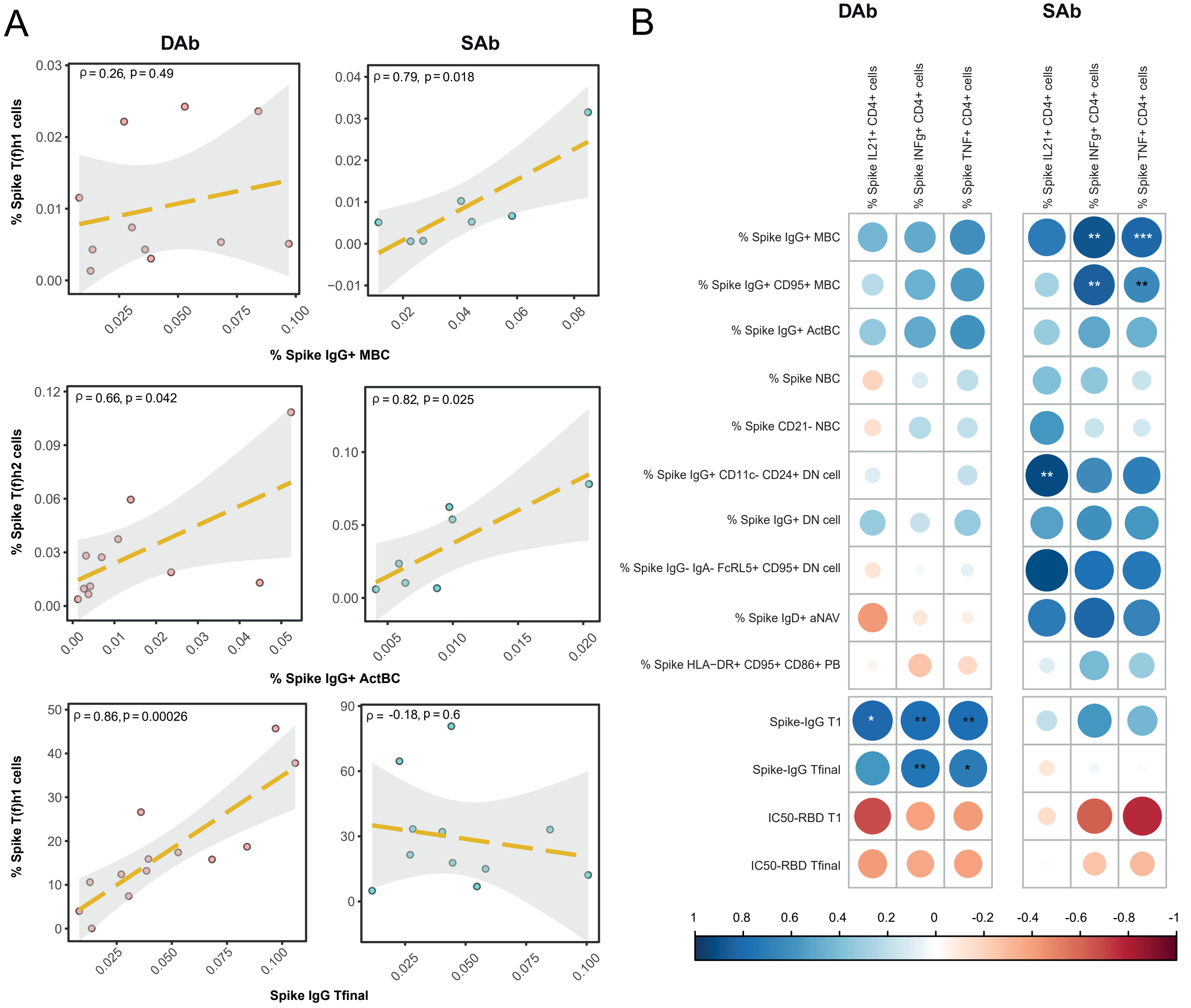

A
DAb
SAb
ρ = 0.26, p = 0.49
ρ = 0.79, p = 0.018
% Spike T(f)h1 cells
% Spike IgG+ MBC
ρ = 0.66, p = 0.042
ρ = 0.82, p = 0.025
% Spike T(f)h2 cells
% Spike IgG+ ActBC
ρ = 0.86, p = 0.00026
ρ = -0.18, p = 0.6
% Spike T(f)h1 cells
Spike IgG Tfinal
B
DAb
SAb
% Spike IL21+ CD4+ cells
% Spike INFg+ CD4+ cells
% Spike TNF+ CD4+ cells
% Spike IgG+ MBC
% Spike IgG+ CD95+ MBC
% Spike IgG+ ActBC
% Spike NBC
% Spike CD21- NBC
% Spike IgG+ CD11c- CD24+ DN cell
% Spike IgG+ DN cell
% Spike IgG- IgA- FcRL5+ CD95+ DN cell
% Spike IgD+ aNAV
% Spike HLA−DR+ CD95+ CD86+ PB
Spike-IgG T1
Spike-IgG Tfinal
IC50-RBD T1
IC50-RBD Tfinal

Table S1. Antibodies used in the spectral flow cytometry analysis of B cells

Target	Fluorochrome	Clone	Source	Catalog No	Dilution
CD11b	PerCP-Cy5.5	M1/70	BioLegend	101227	1/80
CD11c	BV605	B-ly6	BD	563930	1/160
CD138	BUV737	MI15	BD	612834	1/100
CD14	Sparkblue550	63D3	BioLegend	367147	1/80
CD16	BV510	3G8	BioLegend	302047	1/80
CD19	BV570	HIB19	BioLegend	302235	1/40
CD20	eFluor450	2H7	Thermofisher	48-0209-41	1/160
CD21	BUV805	B-ly4	BD	742008	1/160
CD24	BUV395	ML5	BD	566221	1/80
CD27	BV480	L128	BD	566188	1/20
CD3	BV510	UCHT1	BioLegend	300447	1/80
CD38	APC-Fire810	HIT2	BioLegend	303549	1/160
CD4	BV510	OKT4	BioLegend	317443	1/80
CD43	BUV661	1G10	BD	750301	1/160
CD45RB	PE	MEM-55	BioLegend	310204	1/80
CD56	BV510	HCD56	BioLegend	318339	1/80
CD71	BV750	M-A712	BD	747308	1/80
CD73	BV650	AD2	BD	742633	1/80
CD86	APC-R700	2331 (FUN-1)	BD	565149	1/320
CD95	PE-Cy5	DX2	BioLegend	305610	1/320
FcRL4	PerCPeFluor710	413D12	Thermofisher	46-3079-42	1/80
FcRL5	BV785	509F6	BD	749602	1/100
HLA-DR	BUV496	G46-6	BD	749866	1/80
IgA	APC-Vio770	IS11-8E10	Miltenyibiotec	130-113-999	1/320
IgD	BUV563	IA6-2	BD	741394	1/160
IgG	PE-CF594	G18-145	BD	562538	1/160
IgM	BV711	MHM-88	Biolegend	314539	1/80

Table S2. Information on probe fluorophores used in the spectral flow cytometry analysis of B cells

**Fluorochrome-conjugated SA**	**Protein**	**Concentration**	**Source**	**Catalog No**
Alexa Fluor® 647 Streptavidin	S, NC, G	0.5 mg/ml	BioLegend	405237
BB515 Streptavidin	NC, TT	0.1 mg/ml	BD	564453
BUV615 Streptavidin	HA, G	0.1 mg/ml	BD	613013
Briljant Violet 421™ Streptavidin	S, HA, TT	0.1 mg/ml	BioLegend	405226
PE/Cyanine7 Streptavidin	RBD	0.2 mg/ml	eBioscience	25-4317-82

Table S3. Information on antibodies used in the AIM assay to detect and characterize SARS-CoV-2-specific T cells

**Target**	**Fluorochrome**	**Clone**	**Source**	**Catalog No**	**Dilution**
AF647	IL-21	3A3-N2.1	BD	560493	1:50
APC-H7	HLA-DR	G46-6	BD	561358	1:200
APC-R700	IL-17A	N49-653	BD	565163	1:3200
BB515	CCR6 (CD196)	11A9	BD	564479	1:25
BB700	PD1	EH12.1	BD	566460	1:100
BUV395	CD4	SK3	BD	563550	1:50
BUV496	CD8	RPA-T8	BD	612942	1:50
BUV563	CD27	L128	BD	748705	1:400
BUV615	CD14	M5E2	BD	751150	1:100
BUV661	ICOS (CD278)	DX29	BD	741664	1:25
BUV737	CD69	FN56	BD	612817	1:100
BUV805	CD3	SK7	BD	612893	1:200
BV421	CD40L (CD154)	24-31	Biolegend	310824	1:100
BV480	IFNg	B27	BD	566100	1:50
BV605	CCR4 (CD194)	1G1	BD	562906	1:50
BV650	TNF	MAb11	BD	563418	1:400
BV711	CXCR5 (CD185)	RF8B2	BD	740737	1:200
BV750	CD38	HIT2	BD	746909	1:50
BV786	CD45RA	HI100	BD	563870	1:400
FVS575V	viability		BD	565694	1:400
PE	IL-4	8D4-8	BD	559333	1:50
PE-Cy5	CD137 (4-1BB)	4B4-1	BD	551137	1:200
PE-Cy7	CXCR3 (CD183)	1C6	BD	560831	1:25
